# Comparative Studies on the Characteristic Fatty Acid Profiles of Four Different Chinese Medicinal *Sargassum* Seaweeds by GC-MS and Chemometrics

**DOI:** 10.3390/md14040068

**Published:** 2016-03-29

**Authors:** Zhen Chen, Yibing Xu, Tao Liu, Lining Zhang, Hongbing Liu, Huashi Guan

**Affiliations:** 1 Key Laboratory of Marine Drugs, Chinese Ministry of Education, School of Medicine and Pharmacy, Ocean University of China, Qingdao 266003, China; forevercz@gmail.com (Z.C.); kayinamai@163.com (Y.X.); hsguan@ouc.edu.cn (H.G.); 2 Laboratory of Genetics and Breeding of Marine Organisms, College of Marine Life Science, Ocean University of China, Qingdao 266003, China; liutao@ouc.edu.cn; 3 Zhejiang Mariculture Research Institute, Wenzhou 325005, China; zln887766@163.com

**Keywords:** *Sargassum*, fatty acid profile, marine traditional Chinese medicine, chemometrics

## Abstract

*Sargassum* seaweeds produce abundant biomass in China and have long been used as herbal medicine and food. Their characteristic fatty acid (FA) profiles and related potential function in promoting cardiovascular health (CVH) have not been systematically investigated. In this study, FA profiles of four medicinal *Sargassum* were characterized using GC-MS. Principal component analysis was used to discriminate the four medicinal *Sargassum*, and orthogonal projection to latent structures discriminant analysis was carried out between the two official species HAI ZAO and between the two folk medicine species HAI QIAN. In all of the algae investigated, the major SFA and MUFA were palmitic and stearic acid, respectively, while the major PUFAs were linoleic, arachidonic, and eicosapentaenoic acid. *S. fusiforme* and *S. horneri* had higher concentrations of PUFAs. With respect to CVH, all of the studied species, particularly *S. fusiforme*, exhibited satisfactory levels such as PUFA/SFA ratio and *n*-6/*n*-3 ratio. Each species possesses a unique FA profile and is discriminated clearly. Potential key FA markers (between the two Chinese official species, and between the two folk species) are assessed. The study provides characteristic fatty acid profiles of four Chinese medicinal *Sargassum* and their related potential function in promoting CVH.

## 1. Introduction

Polyunsaturated fatty acids (PUFAs) have been shown to have beneficial association with cardiovascular health (CVH) [1]. Several PUFA drugs have been approved by FDA until now, such as Epanova and Lovaza, for treating coronary heart disease and dyslipidemia [2,3]. Being considered as the most important PUFAs supplement, fish oil is a well-known resource of *n*-3 PUFAs, whose substitutes include olive oil, vegetable oil, krill oil, and seaweed oil [4]. It is clear from recent prospective randomized trials that both fish and plant sources of *n*-3 PUFAs can favorably impact CVH [5]. On the other hand, fatty acid (FA) is commonly used as a building-block of complex lipids, such as glycoglycerolipids. We found that monogalactosyldiacylglycerols (MGDGs) from marine seaweed *Sargassum horneri* showed significant effects on triglyceride accumulation inhibition in 3T3-L1 adipocytes [6].

*Sargassum*, a genus of brown seaweed (Phaeophyceae) in the Fucales order, is abundant in China. Some species in this genus have been widely used as traditional Chinese medicine (TCM) for thousands of years. *S. fusiforme* (*Hizikia* *fusiforme*) and *S. pallidum* are the two official species recorded in Chinese Pharmacopoeia and are both referred to as “HAI ZAO”. *S. horneri* and *S. thunbergii* are used as folk medicine and are both referred to as “HAI QIAN”. These four seeweeds possess a similar function and usage, for treating atherosclerosis, hyperlipidemia, hypertension, thyroid diseases, and cancer [7,8]. *Sargassum* algae are rich in fatty acids, which are known to play an important role in CVH. However, the characteristic fatty acid profiles of these marine seaweeds TCM and their function in improving CVH have not been systematically investigated.

The objective of this study was to characterize the fatty acid profiles of four medicinal *Sargassum* (*S. fusiforme*, *S. pallidum*, *S. horneri*, and *S. thunbergii*) in China via gas chromatography-mass spectrometry (GC-MS). Their potential effects on CVH were evaluated by nutritional indices, such as the *n*-6 PUFA/*n*-3 PUFA ratio [9], the PUFA/SFA ratio [10], the unsaturation index (UI), the atherogenic index (AI), and the thrombogenic index (TI) [11]. The characteristic FAs for these species were evaluated using chemometric tool analysis. It is known that FA composition varies along with environmental factors, which make it more complicated [12]. Thus, large-scale representative samples are required for the characteristic FA profile of individual alga. Detailed comparison between the two pharmacopoeia species (*S. fusiforme* and *S. pallidum*) and between the two folk medicine species (*S. horneri*, and *S. thunbergii*) were constructed.

## 2. Results and Discussion

### 2.1. Total Lipids

**Figure 1:**
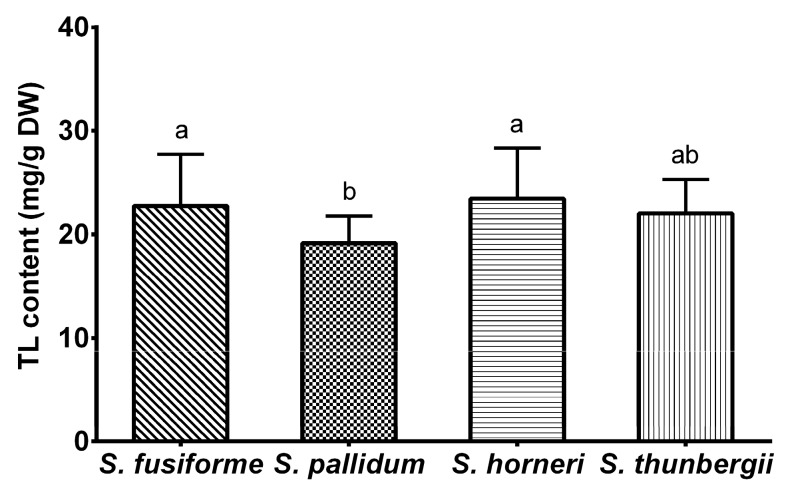
Total lipid contents of four *Sargassum* algae. The data are presented as means ± SD. Different letters (a,b) indicate significant differences by the Tukey *post hoc* test (*p* < 0.01).

The total lipid (TL) contents in the four algae investigated in this study are shown in Figure 1. *S. horneri* exhibited the highest TL content (23.4 ± 4.9 mg/g DW), which was lower than that reported by Nomura (47.4 ± 0.8–142.5 ± 32.0 mg/g DW) [13] and Terasaki (62.6 ± 18.7 mg/g DW) [14] but higher than that reported by Murakami (5.17 ± 0.17–11.5 ± 0.7 mg/g DW) [15]. The TL contents of *S. fusiforme* (22.7 ± 5.0 mg/g DW) and *S. thunbergii* (22.0 ± 3.3 mg/g DW) were in accordance with the literature (15.7–27.5 mg/g DW for *S. fusiforme* [14,16,17] and 31.8 ± 13.1 mg/g DW for *S. thunbergii* [14]). The lowest value was observed in *S. pallidum* (18.2 ± 1.6 mg/g DW), which was significantly different from both *S. horneri* and *S. fusiforme* (*p* < 0.01) in the present study.

### 2.2. Fatty Acid Profiles

**Figure 2:**
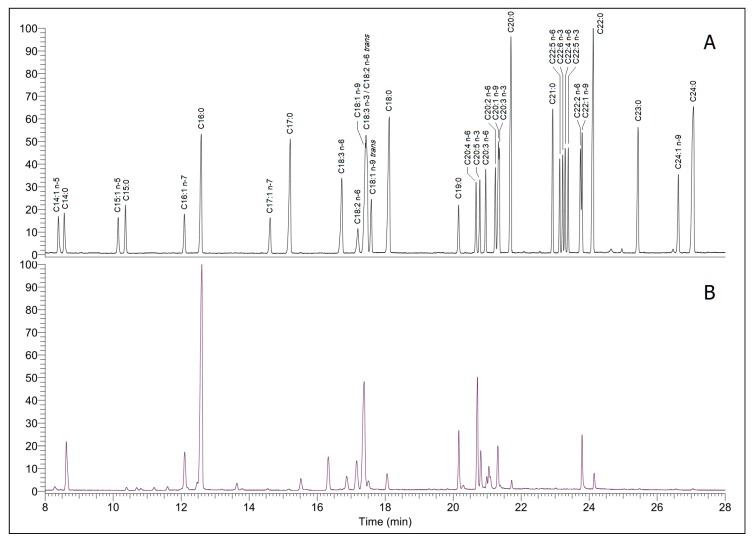
The total ion chromatograms of the standard FAMEs mixture and *Sargassum* algal sample. (**A**) Standard FAMEs mixture; (**B**) Sample of *S. fusiforme* 1.

The fatty acid contents (composition/profiles) and nutritional indices related to CVH of the four species are presented in Table 1. The four algal species exhibited similar FA patterns but differed significantly in their FA contents (*p* < 0.05 for all FAs). Typical GC-MS chromatogram is shown in Figure 2.

Within the algae analyzed, the saturated fatty acids (SFAs) varied from 41.70% ± 6.88% of total fatty acid methyl esters (FAMEs) in *S. fusiforme* to 61.12% ± 4.80% in *S. pallidum*. Monounsaturated fatty acids (MUFAs) ranged from 27.09% ± 1.18% of total FAME in *S. pallidum* to 32.89% ± 1.07% in *S. thunbergii*. In all the species, palmitic acid (C16:0) was the dominant FA, and oleic acid (C18:1 *n*-9) was the dominant unsaturated fatty acid (UFA). These findings are in agreement with previous studies, in which C16:0 was the major FA in all *Sargassum* species [18,19,20].

*S. fusiforme* was the species with the highest PUFA levels (26.09% ± 7.97%); *S. pallidum*, in contrast, had the lowest PUFA levels (11.79% ± 4.62%). For the *n*-3 PUFA family, the highest proportion was also found in *S. fusiforme* (8.58% ± 4.37%), and the lowest proportion was observed in *S. pallidum* (1.71% ± 0.95%). Eicosapentaenoic acid (C20:5 *n*-3, EPA), stearidonic acid (18:4 *n*-3, SDA), and eicosatetraenoic acid (C20:4 *n*-3, ETA) were the only three n-3 PUFAs detected. The *n*-6 PUFA family content varied from 10.07% ± 3.70% in *S. pallidum* to 19.91% ± 1.78% in *S. horneri*. The most abundant *n*-6 PUFA was arachidonic acid (C20:4 *n*-6, ARA) in *S. fusiforme* (9.37% ± 3.38%) and *S. horneri* (10.70% ± 1.19%) and linoleic acid (C18:2 *n*-6, LNA) in *S. pallidum* (5.03% ± 1.66%) and *S. thunbergii* (7.01% ± 0.97%). Our observation that ARA and LNA comprised the major *n*-6 PUFAs in the algae we investigated is in keeping with other reports on *S. fusiforme* [14,16,20], *S. pallidum* [19,21], *S. horneri* [13,14,22], and *S. thunbergii* [14,20,23,24]. In addition, minor amounts of γ-linolenic acid (C18:3 *n*-6, GLA), eicosadienoic acid (C20:2 *n*-6), and dihomo-γ-linolenic acid (C20:3 *n*-6, DGLA) were recorded.

It should be noted that lower levels of PUFAs were recorded in our study than in other studies. Dawczynski [15] found much higher levels of EPA in fresh *S. fusiforme* (42.4% ± 11.88%), which was approximately 10-fold greater than our measurement (4.29% ± 2.10%). In addition, the ARA content was reported to be 15.5%–38.5% in *S. pallidum* [19,25,26] and 10.2%–14.8% in *S. thunbergii* [14,20,23], and a high content of DGLA (14.5%–16.5%) in *S. pallidum* was reported as well [26]. Khotimchenko demonstrated that different parts of the thallus of *S*. *pallidum* differ in the content of DGLA and other PUFAs, and the distribution of PUFAs within the thallus does not depend on environmental factors [18]. Sanina posited the opposite result, that unsaturation and the *n*-3/*n*-6 ratio of FAs in the glycolipids of *S. pallidum* rise from summer to winter [12]. In our opinion, aside from environmental factors, the algal material drying process is primarily responsible for the loss of PUFA content [6,27,28]. In the present study, the cleaned algae were dried under sunlight and stored in the dark at room temperature, which follows the processing method of food and herbal medicine in China. There are several studies on FAs of *S. fusiforme* in China that performed a similar processing method. The literature data showed 5.8%–11.8% for C18:2 *n*-6, 0.7%–3.3% for C18:3 *n*-3, 0.5%–16.4% for C20:4 *n*-6, and 4.4%–7.9% for C20:5 *n*-3 [29,30,31]. Our results are consisted with the literature.

The PUFA/SFA ratio is a primary indicator used to evaluate the lipid quality, and its recommended minimum value is 0.45 by the British Department of Health [10]. That level is exceeded by the PUFA/SFA ratios found in *S. fusiforme* (0.67) and *S. horneri* (0.56) in the present study.

The lipid contents of marine algae are unique with respect to their low ratio of *n*-6 to *n*-3 polyunsaturated fatty acids (PUFA) [32]. The balance between *n*-6 and *n*-3 PUFAs is demonstrated in several studies to associate with an improvement in whole body glucose tolerance, obesity, inflammatory, and other metabolic dysfunction [33]. The *n*-6 PUFA/*n*-3 PUFA ratio should not exceed 10 according to WHO recommendations [9], while the European Nutritional Societies suggest this ratio not exceed 5 for the prevention of inflammatory, cardiovascular, and neurological disorders [34]. All the examined *Sargassum* species exhibited a favorable *n*-6/*n*-3 ratio below 5—except *S. pallidum* (7.17).

The artherogenic index (AI) and thrombogenic index (TI) are proposed to evaluate risky factors that are implicated in coronary heart disease development by Ulbricht [11]. Both of them were <2 in the investigated species, owing to the high *n*-3 PUFA contents and low *n*-6/*n*-3 ratios. The results suggest that the *Sargassum* might be beneficial to cardiovascular health.

### 2.3. Principal Component Analysis

**Figure 3:**
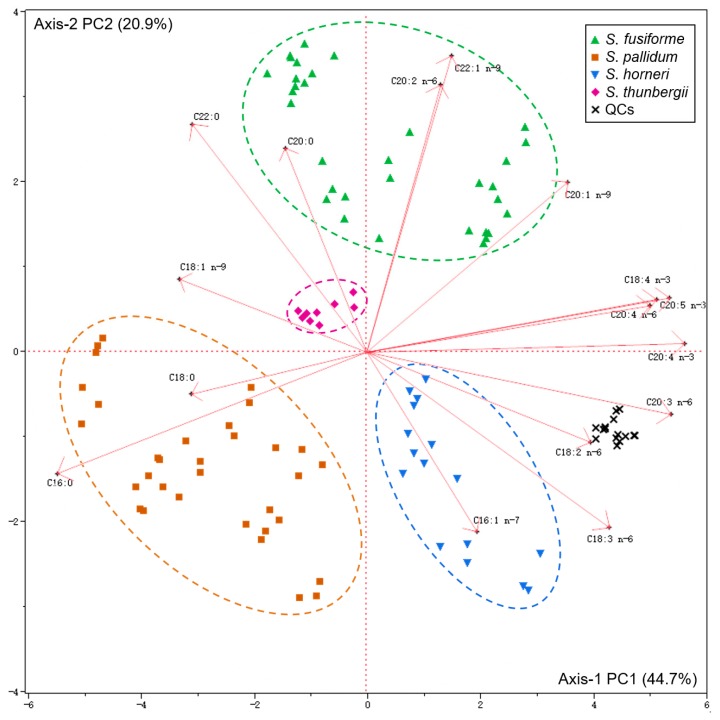
Bi-plot of *Sargassum* samples obtained by the PCA of FA variables.

A principal component analysis (PCA) was performed, and a bi-plot graph (scores plot and loading plot) was obtained using the FA variables (Figure 3). The first two principal components (PC1 and PC2) accounted for 44.7% and 20.9% of the total variation, respectively. Generated from comparisons of the first two PCs, the bi-plots revealed four distinct groups of samples. The QC samples clustered together in the plot, which verifies the reliability of the GC-MS data [35,36]. Clear separation of the algal samples was observed, and no outliers were found, which indicates that the fatty acid profiles were quite different among the studied species.

Specific patterns of correlation between PCs and FA variables are visualized in Figure 3. Axis-1 represents the degree of unsaturation, which indicates that algae with high UFA contents (particularly *n*-3 and *n*-6 PUFAs) are oriented in the positive axis-1, whereas those algae with higher amounts of SFAs are centered more towards the negative axis-1. Axis-2 expresses the length of the FA carbon chains. As the value on axis-2 rises, the lengths of the FA carbon chains in the algae increase (Figure 3). A closer examination of the PCA results indicated that *S. pallidum* was correlated with stearic acid (C18:0) and palmitic acid (C16:0), whereas *S. fusiforme* showed a strong positive correlation with C20 and C22 FAs. Moreover, *S. horneri* had higher positive loadings for C18 *n*-6 PUFAs (including LNA and GLA). A weak positive correlation with FAs was observed in *S. thunbergii.*

Our results revealed that each individual species possesses a unique FA profile. FA signature is considered as potential chemotaxonomic tool for closer understanding the relationships among different macroalgae species at ordinal levels of families, orders, and phyla [18]. Poor evidence is found for FA signature applying to classify different species in *Sargassum* genus until now. Therefore, a PCA of FA profiles may be a potential chemotaxonomic tool for *Sargassum* species.

### 2.4. Orthogonal Projection to Latent Structures Discriminant Analysis

**Figure 4:**
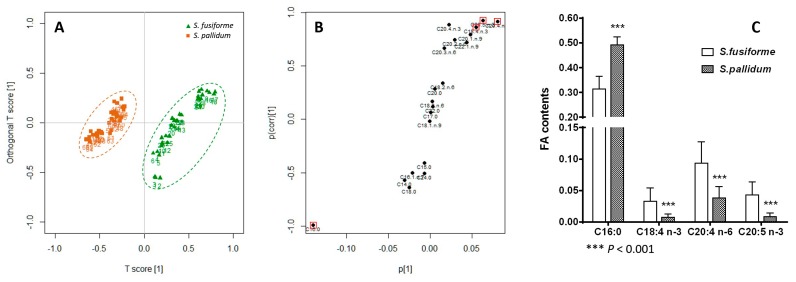
OPLS-DA of the two official species HAI ZAO (*S. fusiforme* and *S. pallidum*). (**A**) Score scatter plot; (**B**) S-plot; (**C**) Potential key FA contents.

*S. fusiforme* and *S. pallidum* are the two official species HAI ZAO with the same function and usage in Chinese Pharmacopoeia. In the present study, their FA profiles were significantly different (Table 1), particularly with respect to nutritional indices such as the *n*-6/*n*-3 ratio, AI, and TI. To reveal potential key FAs differences between them, orthogonal projection to latent structures discriminant analysis (OPLS-DA) was applied to the FA profiles. The score scatter plot and S-plot were generated after Pareto scaling and mean-centering. In score scatter plot (Figure 4A), *S. fusiforme* and *S. pallidum* were highly discriminated by OPLS-DA. In S-plot (Figure 4B), the *x*-axis represents the contribution of a particular marker to the group differences, while the Y-axis denotes confidence of the marker’s contribution. All the long chain (C20 and C22) PUFAs were grouped in the upper-right quadrant, which displayed the increased FAs in *S. fusiforme*. Meanwhile, most of the SFA were in the lower-left quadrant, as they were *S. fusiforme*’s decreased FAs. The top four important variables were selected according to the variable importance in the projection (VIP) scores and marked in the S-plot. As shown in Figure 4C, *S. fusiforme* exhibited significantly higher contents of PUFAs C18:4 *n*-3, C20:4 *n*-6, and C20:5 *n*-3 than *S. pallidum*. In contrast, the content of C16:0, the only SFA detected, was distinctly lower in *S. fusiforme* (*p* < 0.001 for all the FAs). There were obvious differences in the aspect of FA profile between *S. fusiforme* and *S. pallidum* from the current results.

**Figure 5:**
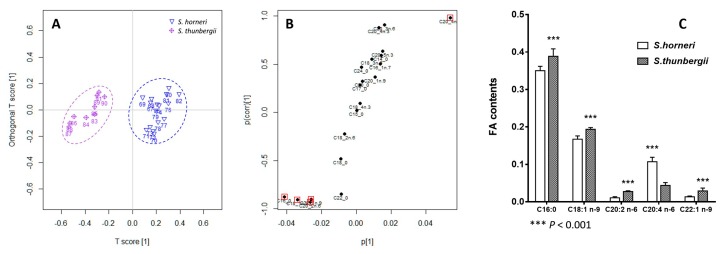
OPLS-DA of the two folk medicine species HAI QIAN (*S. horneri* and *S. thunbergii*). (**A**) Score scatter plot; (**B**) S-plot; (**C**) Potential key FA contents.

Similarly, OPLS-DA was performed to discern the separation between the two folk medicine HAI QIAN, *S. horneri* and *S. thunbergii*. They were clearly discriminated as well in the score scatter plot (Figure 5A). However, the FA variables distributed irregularly in the S-plot (Figure 5B), which was different from that in the two official species HAI ZAO. Nevertheless, top five important variables were found, namely, C16:0, C18:1 *n*-9, C20:2 *n*-6, and C22:1 *n*-9 for the increased FAs in *S. thunbergii*, and C20:4 *n*-6 increased in *S. horneri* (Figure 5C).

## 3. Experimental Section

### 3.1. Sampling and Processing of Algal Material

In total, the 91 algae samples used in this study were collected from the east coast of China: 33 samples of *S. fusiforme*, 33 samples of *S. pallidum*, 16 samples of *S. horneri*, and 9 samples of *S. thunbergii* (see Table S1). All the algal materials used in this study were provided by the Culture Collection of Seaweed at the Ocean University of China. The collected algae were identified by Prof. Dr. Tao Liu (College of Marine Life Science, Ocean University of China), and voucher specimens have been deposited at the School of Medicine and Pharmacy, Ocean University of China. The collected algal materials were washed with fresh water followed by distilled water and thoroughly cleaned of epiphytes. In accordance with the drying processes used for food and herbal medicine in daily life in China, the cleaned algae were dried under sunlight instead of freeze-drying. The dried fronds were cut into short pieces and stored in the dark at room temperature until use.

### 3.2. Lipid Extraction

The dried algae were ground into 40-mesh powders before analysis. The Bligh and Dyer method was performed for total lipid extraction [37]. Briefly, approximately 750 mg of powder was extracted with chloroform:methanol (1:2 *v*/*v*) containing BHT (0.01% *w*/*v*) and then evaporated to dryness in vacuum. The extracted lipids were weighed, and subsequent analyses were performed immediately.

### 3.3. Fatty Acid Esterification

The fatty acids were converted into their corresponding FAMEs via transmethylation of the lipid samples with 1.0 mL of 1 M KOH in anhydrous methanol, followed by heating for 10 min at 70 °C. Subsequently, 2.0 mL of 1 M methanolic HCl were added, followed by heating for 10 min at 70 °C and the addition of 1 mL of water [38]. Nonadecanoic acid (C19:0) was used as an internal standard, and the FAMEs were extracted in hexane.

### 3.4. Fatty Acid Analysis

GC-MS analysis was performed using a Thermo Trace 1300 ISQ gas chromatography-mass spectrometer (Thermo Fisher Scientific Inc., San Jose, CA, USA) equipped with a TR5-MS capillary column of 30 m × 0.25 mm × 0.25 μm (Thermo Fisher Scientific Inc., San Jose, CA, USA). The column temperature was initially set at 120 °C, held at that temperature for 1 min after injection, increased at a rate of 15 °C/min to 180 °C, and held at that temperature for 1 min. Next, the temperature was continually increased at a rate of 2 °C/min to 200 °C, held at that temperature for 1 min, and then increased at a rate of 10 °C/min to 260 °C, followed by a final hold at that temperature for 1 min. The injection was performed in the splitless mode, and the injection temperature was set at 250 °C. Nitrogen (99.9% purity) was used as the carrier gas at a flow rate of 1 mL/min. The interface temperature was set at 250 °C and the electron energy was 70 eV. The scan range was from *m/z* 20 to 500 in mass detection conditions, and the chromatograms were acquired in full scan mode. All analyses were performed in triplicate. Visualization, calibration, and format transfer of the GC-MS data were performed using Xcalibur 2.1 (Thermo Fisher Scientific Inc.), followed by deconvolution using IXCR (Advanced Chemistry Development, Inc., Toronto, ON, Canada). The FAME standards (purity: 99%) mixture was purchased from Nu-Chek-Prep, Inc. (Elysian, MN, USA). The FAME peaks were identified using the NIST 11 MS data library and a comparison of their retention times with authentic standards. All the FAME peaks were quantified by area normalization with threshold set at 0.1% prior to data analysis and statistics.

### 3.5. Quality Control

The data acquisition sequence was randomized to minimize the time variation, as in the GC-MS experiment. Quality control (QC) samples, consisting of a representative average of samples pooled from different final sample extracts, were tested at the beginning, end, and randomly throughout the analytical run to ensure the stability of the GC-MS method [35,39].

### 3.6. Statistical Analysis

GraphPad Prism 6.0 was used for statistical analysis. All data are presented as the means ± standard deviation (SD). Statistical analysis was conducted using the unpaired two-tailed *t*-test and one-way ANOVA (using the Tukey *post hoc* test). *p* < 0.05 was considered to be statistically significant. JMP 10.0 (SAS Institute Inc., Cary, NC, USA) and R-package muma [40] used for multivariate statistical analysis.

## 4. Conclusions

In this study, FA profiles of four medicinal *Sargassum* seaweeds (*S. fusiforme*, *S. pallidum*, *S. horneri*, and *S. thunbergii*) were characterized using GC-MS. Each species possesses unique FA profile characteristics, as assessed using PCA. Potential key FAs comprising significant differences between the two Chinese official species, and between the two folk medicine species, were assessed using OPLS-DA, respectively. With respect to cardiovascular health, all of the studied species, particularly *S. fusiforme*, exhibit satisfactory nutritional indices, such as the PUFA/SFA ratio, the *n*-6 PUFA/*n*-3 PUFA ratio, AI, and TI. Our current study suggests that the *Sargassum* might be beneficial to CVH.

## Figures and Tables

**Table 1:** Fatty acid profiles (given in % of total FAME) and nutritional indices related to CVH in different *Sargassum* species (expressed as means ± SD).

Fatty Acid (%)	*S. fusiforme*	*S. pallidum*	*S. horneri*	*S. thunbergii*
C14:0	5.33 ± 1.22 ^b^	6.70 ± 1.73 ^a^	5.90 ± 0.55 ^a,b^	5.23 ± 0.53 ^a,b^
C15:0	0.51 ± 0.14 ^a,b^	0.58 ± 0.11 ^a^	0.42 ± 0.11 ^b^	0.41 ± 0.08 ^b^
C16:0	31.44 ± 5.07 ^c^	48.66 ± 3.69 ^a^	35.02 ± 1.17 ^b^	38.86 ± 1.99 ^b^
C17:0	0.28 ± 0.10 ^a^	0.25 ± 0.05 ^b^	0.20 ± 0.05 ^b^	0.16 ± 0.07 ^b^
C18:0	1.95 ± 0.68 ^b^	2.79 ± 0.69 ^a^	1.65 ± 0.32 ^b^	2.02 ± 0.33 ^b^
C20:0	0.86 ± 0.17 ^a^	0.80 ± 0.10 ^b^	0.71 ± 0.08 ^b^	0.68 ± 0.18 ^b^
C22:0	1.15 ± 0.30 ^a^	1.07 ± 0.21 ^b^	0.64 ± 0.07 ^c^	0.84 ± 0.05 ^b,c^
C24:0	0.18 ± 0.09 ^b^	0.28 ± 0.05 ^a^	0.17 ± 0.05 ^b,c^	0.11 ± 0.01 ^c^
∑SFA	41.70 ± 6.88 ^c^	61.12 ± 4.80 ^a^	44.71 ± 1.41 ^b,c^	48.30 ± 1.83 ^b^
C16:1 *n*-7	4.34 ± 0.98 ^c^	5.19 ± 0.70 ^b^	7.15 ± 0.88 ^a^	6.41 ± 0.37 ^a^
C18:1 *n*-9	18.91 ± 1.49 ^a^	19.57 ± 1.38 ^a^	16.71 ± 0.88 ^b^	19.36 ± 0.47 ^a^
C20:1 *n*-9	4.57 ± 0.82 ^a,b^	1.19 ± 0.31 ^c^	5.06 ± 1.04 ^a^	4.21 ± 0.20 ^b^
C22:1 *n*-9	4.39 ± 1.13 ^a^	1.15 ± 0.26 ^c^	1.35 ± 0.19 ^c^	2.92 ± 0.75 ^b^
∑MUFA	32.21 ± 1.91 ^a^	27.09 ± 1.18 ^c^	30.27 ± 1.44 ^b^	32.89 ± 1.07 ^a^
C18:2 *n*-6	4.89 ± 0.63 ^b^	5.03 ± 1.66 ^b^	6.59 ± 0.99 ^a^	7.01 ± 0.97 ^a^
C18:3 *n*-6	0.26 ± 0.08 ^b^	0.26 ± 0.14 ^b^	0.57 ± 0.31 ^a^	0.29 ± 0.09 ^b^
C18:4 *n*-3	3.33 ± 2.11 ^a^	0.52 ± 0.33 ^b^	1.52 ± 0.64 ^b^	1.50 ± 0.53 ^b^
C20:2 *n*-6	2.19 ± 0.49 ^b^	0.91 ± 0.23 ^c^	1.07 ± 0.30 ^c^	2.75 ± 0.20 ^a^
C20:3 *n*-6	0.81 ± 0.31 ^a^	0.55 ± 0.31 ^b^	0.98 ± 0.21 ^a^	0.38 ± 0.15 ^b^
C20:4 *n*-6	9.37 ± 3.38 ^a^	3.33 ± 1.53 ^b^	10.70 ± 1.19 ^a^	4.37 ± 0.77 ^b^
C20:4 *n*-3	0.96 ± 0.28 ^a^	0.49 ± 0.22 ^b^	0.93 ± 0.07 ^a^	0.55 ± 0.24 ^b^
C20:5 *n*-3	4.29 ± 2.10 ^a^	0.71 ± 0.43 ^c^	2.66 ± 0.53 ^b^	1.94 ± 0.46 ^b^
∑PUFA	26.09 ± 7.97 ^a^	11.79 ± 4.62 ^c^	25.03 ± 2.10 ^a^	18.81 ± 1.88 ^b^
PUFA/SFA	0.67 ± 0.31 ^a^	0.20 ± 0.09 ^c^	0.56 ± 0.06 ^a,b^	0.39 ± 0.05 ^b,c^
∑*n*-6 FA	17.51 ± 3.72 ^a,b^	10.07 ± 3.70 ^c^	19.91 ± 1.78 ^a^	14.81 ± 0.88 ^b^
∑*n*-3 FA	8.58 ± 4.37 ^a^	1.71 ± 0.95 ^c^	5.11 ± 0.99 ^b^	4.00 ± 1.20 ^b,c^
*n*-6/*n*-3	2.43 ± 0.83 ^c^	7.17 ± 2.75 ^a^	4.03 ± 0.80 ^b^	3.98 ± 1.11 ^b,c^
UI ^A^	125.65 ± 32.25 ^a^	62.27 ± 15.05 ^c^	116.16 ± 5.77 ^a^	89.87 ± 7.44 ^b^
AI ^B^	0.94 ± 0.28 ^b^	1.99 ± 0.45 ^a^	1.06 ± 0.06 ^b^	1.16 ± 0.10 ^b^
TI ^C^	0.46 ± 0.21 ^b^	1.60 ± 0.56 ^a^	0.65 ± 0.07 ^b^	0.76 ± 0.14 ^b^

^a–c^ Values in a row without a common superscript were significantly different at the 0.05 probability level; ^A^ UI (unsaturation index) was calculated by multiplying the percentage of each fatty acid by the number of double bonds, followed by summing up these contributions; ^B^ AI (atherogenic index): AI = [C12:0 + (4 × C14:0) + C16:0]/(∑MUFAs + *n*-3 PUFAs + *n*-6 PUFAs); ^C^ TI (thrombogenic index): TI = (C14:0 + C16:0 + C18:0)/[(0.5 ×∑MUFAs) + (0.5 × *n*-6 PUFAs) + (3 × *n*-3 PUFAs) + (*n*-3 PUFAs/*n*-6 PUFAs)].

## Supplementary Materials

The following are available online at www.mdpi.com/1660-3397/14/4/68/s1, Table S1: Algal samples information.

## Abbreviations

The following abbreviations are used in this manuscript:

TCM	traditional Chinese medicine
CVH	cardiovascular health
FA	fatty acid
FAME	fatty acid methyl ester
SFA	saturated fatty acid
UFA	unsaturated fatty acid
MUFA	monounsaturated fatty acid
PUFA	polyunsaturated fatty acid
UI	unsaturation index
AI	atherogenic index
TI	thrombogenic index
PCA	principal component analysis
OPLS-DA	orthogonal projection to latent structures discriminant analysis
VIP	variable importance in the projection
QC	quality control
