# Supplementary material for: Comparative Studies on the Characteristic Fatty Acid Profiles of Four Different Chinese Medicinal Sargassum Seaweeds by GC-MS and Chemometrics
Source: Mar Drugs. 2016 Mar 29;14(4):68. doi: 10.3390/md14040068 (PMC4849072; doi:10.3390/md14040068)
Supplement: Supplementary file 1 [file marinedrugs-14-00068-s001.pdf]

Supplementary Materials: Comparative Studies on the Characteristic Fatty Acid Profiles of Four Different Chinese Medicinal *Sargassum* Seaweeds by GC-MS and Chemometrics

Zhen Chen, Yibing Xu, Tao Liu, Lining Zhang, Hongbing Liu and Huashi Guan

Table S1. Algal samples information.

		Growing Region	Collection Time
S. fusiforme			
	1	Zhejiang	March 2012
	2	Zhejiang	March 2012
	3	Zhejiang	March 2012
	4	Zhejiang	May 2012
	5	Zhejiang	May 2012
	6	Zhejiang	May 2012
	7	Shandong	May 2013
	8	Shandong	May 2013
	9	Shandong	May 2013
	10	Zhejiang	May 2013
	11	Zhejiang	May 2013
	12	Zhejiang	May 2013
	13	Zhejiang	April 2012
	14	Zhejiang	April 2012
	15	Zhejiang	April 2012
	16	Zhejiang	May 2013
	17	Zhejiang	May 2013
	18	Zhejiang	May 2013
	19	Shandong	August 2011
	20	Shandong	August 2011
	21	Shandong	August 2011
	22	Shandong	August 2012
	23	Shandong	August 2012
	24	Shandong	August 2012
	25	Zhejiang	April 2013
	26	Zhejiang	April 2013
	27	Zhejiang	April 2013
	28	Shandong	April 2013
	29	Shandong	April 2013
	30	Shandong	April 2013
	31	Shandong	April 2013
	32	Shandong	April 2013
	33	Shandong	April 2013
S. pallidum			
	34	Shandong	2012
	35	Shandong	2012
	36	Shandong	2012
	37	Shandong	2012
	38	Shandong	2012
	39	Shandong	2012
	40	Shandong	2012
	41	Fujian	2012
	42	Fujian	2012

	43	Fujian	2012
	44	Fujian	2012
	45	Fujian	2012
	46	Fujian	2012
	47	Fujian	2012
	48	Fujian	2012
	49	Fujian	2012
	50	Fujian	2012
	51	Fujian	Unknown
	52	Fujian	Unknown
	53	Fujian	Unknown
	54	Shandong	September 2013
	55	Shandong	September 2013
	56	Shandong	September 2013
	57	Shandong	September 2013
	58	Shandong	September 2013
	59	Shandong	September 2013
	60	Shandong	September 2013
	61	Shandong	September 2013
	62	Shandong	September 2013
	63	Shandong	September 2013
	64	Fujian	September 2013
	65	Fujian	September 2013
	66	Fujian	September 2013
S. horneri			
	67	Zhejiang	May 2012
	68	Zhejiang	May 2012
	69	Zhejiang	May 2012
	70	Zhejiang	May 2012
	71	Zhejiang	March 2012
	72	Zhejiang	March 2012
	73	Zhejiang	March 2012
	74	Shandong	August 2012
	75	Shandong	August 2012
	76	Shandong	August 2012
	77	Shandong	October 2013
	78	Shandong	October 2013
	79	Shandong	October 2013
	80	Unknown	October 2013
	81	Unknown	October 2013
	82	Unknown	October 2013
S. thunbergii			
	83	Shandong	October 2013
	84	Shandong	October 2013
	85	Shandong	October 2013
	86	Shandong	October 2013
	87	Shandong	October 2013
	88	Shandong	October 2013
	89	Unknown	2011
	90	Unknown	2011
	91	Unknown	2011
